# Boron nutrition in horticultural crops for enhancing yield and fruit quality

**DOI:** 10.3389/fpls.2026.1951662

**Published:** 2026-10-23

**Authors:** D. Rajani, Bekkam Rakesh, N. Rathna Kumari

**Affiliations:** 1 Department of Horticulture, Professor Jayashankar Telangana Agricultural University, Hyderabad, India; 2 Deparment of Soil Science and Agricultural Chemistry, Professor Jayashankar Telangana Agricultural University, Hyderabad, India; 3 Department of Horticulture, Acharya N.G. Ranga Agricultural University, Bapatla, India

**Keywords:** crop-specific responses, dose-dependent response, horticulture crops, nano-boron, post-harvest quality

## Abstract

Boron (B) is an essential micronutrient involved in reproductive development, fruit growth, yield formation, and quality of horticultural crops. It contributes to cell wall integrity, membrane function, carbohydrate transport, pollen germination and pollen tube growth, fruit set, and nutrient translocation. B availability influences reproductive development, yield, and fruit quality, with both deficiency and excess adversely affecting crop performance, while optimal B nutrition can promote fruit development and quality. However, B responses vary considerably with crop species and cultivar, initial B status, application rate and source, and environmental conditions. This review critically examines the physiological and biochemical roles of B and the proposed mechanisms underlying its effects in horticultural crops, with emphasis on reproductive development, yield, fruit quality, and postharvest performance. It evaluates variability and limitations in the available evidence, particularly dose- and crop-dependent responses, and critically assesses the emerging use of nano-boron, including concerns related to phytotoxicity, environmental safety, nanoparticle stability, economic feasibility, and limited field validation. Although nano-boron has shown promising potential, current evidence remains insufficient to establish its consistent superiority over conventional B sources across horticultural crops. Key research gaps and priorities for developing safe, effective, and field-applicable B management strategies are also identified.

## 1 Introduction

Imbalanced fertilization can cause physiological disorders, increase susceptibility to pests and diseases, and reduce fruit quality (Fischer et al., 2021). Boron (B) warrants particular attention because its optimal range lies between relatively narrow deficiency and toxicity limits. Both insufficient and excessive B can impair reproductive development, cellular integrity, and metabolism, affecting fruit growth, yield, and quality. Moreover, B requirements and responses vary among crops and cultivars, depending on B status, dose, source, application timing, and environmental conditions. Therefore, B management requires crop- and cultivar-specific approaches. Boron performs diverse structural, physiological, and metabolic functions essential for plant growth and productivity (Al-Abbasi et al., 2026).

Structurally, boron maintains cell wall integrity through borate–pectin complexes, particularly dRG-II–borate dimers, and contributes to membrane integrity and permeability (Long and Peng, 2023; Mertoglu et al., 2026). Adequate B nutrition may therefore support fruit firmness, structural integrity, postharvest weight retention, fruit set, growth, and productivity through its roles in cellular integrity, reproductive development, and assimilate partitioning (Pandey et al., 2026; Bhantana et al., 2026; Zhang et al., 2024). Conversely, inadequate B supply can impair cell wall development and has been associated with fruit cracking and altered fruit quality in several horticultural crops (Parthiban et al., 2021; Seo et al., 2022). Foliar B application, alone or in combination with Ca, has also been reported to improve fruit firmness and postharvest quality in some fruits, including Asian pear.

Physiologically, boron is essential for reproductive development, including pollen germination, pollen tube growth, fertilization, fruit set, and retention (Hapuarachchi et al., 2022). It also contributes to carbohydrate transport and assimilate partitioning; however, B mobility and redistribution vary among species, and its effects should therefore be interpreted in a crop-specific context. B deficiency can directly impair reproductive development and cause disorders such as corky tissue formation and internal breakdown. Its association with other disorders, such as blossom-end rot, may involve interactions with calcium nutrition and should therefore be considered indirect rather than a direct consequence of B deficiency (Vera-Maldonado et al., 2024). Metabolically, boron influences carbohydrate and organic acid metabolism, secondary metabolism, and antioxidant responses, with effects varying according to crop, tissue, developmental stage, and B status. B deficiency is associated with altered carbohydrate partitioning, organic acid metabolism, oxidative stress, phenolic accumulation, and changes in lignin and phenyl propanoid metabolism (Dong et al., 2022). In fruit crops, B nutrition may also affect soluble solids, acidity, ascorbic acid, pigments, phenolics, and other quality attributes, although responses vary among crops and growing conditions. However, the magnitude and direction of B responses vary considerably among horticultural crops and cultivars, depending on plant B status, application rate, source, timing, and growing conditions. Thus, although several physiological functions of B are well established, responses related to fruit development and biochemical quality are not uniform, and their underlying mechanisms require further critical evaluation.

In recent years, nano-boron has emerged as a promising approach for improving B delivery and utilization in horticultural crops. The nanoscale characteristics of nano-B formulations may influence nutrient absorption, translocation, and utilization; however, the extent to which these properties improve nutrient-use efficiency compared with conventional B formulations remains insufficiently established. Studies have reported beneficial effects of nano-B on growth, yield, fruit quality, and nutrient status; however, the available evidence is not yet sufficient to establish that nano-B consistently performs better than conventional B sources. Differences in formulation, particle characteristics, application rate, application method, crop species, cultivar, and experimental conditions complicate direct comparisons among studies. Therefore, the potential advantages of nano-B should be evaluated alongside its limitations, including the need for appropriate dose optimization and greater evidence concerning its efficacy, safety, and long-term applicability. Despite the established importance of B in horticultural production, responses to B application remain variable and depend on crop species and cultivar, initial B status, source and dose, application timing and method, and environmental conditions. Moreover, evidence for nano-B remains comparatively limited, with substantial variation in nanoparticle characteristics and experimental approaches and relatively few direct comparisons with conventional B. Therefore, the present review critically evaluates the role of B and nano-B in horticultural crops, with emphasis on established and proposed mechanisms, crop- and dose-dependent responses, areas of consensus and contradiction, and limitations and research gaps related to yield, fruit quality, postharvest performance, safety, and practical application.

## 2 Methodology

This review was conducted as a narrative critical synthesis supported by a structured literature search and selection process. Relevant literature on boron nutrition and its effects on horticultural crops was identified using the database (Scopus). Scopus was selected because its multidisciplinary scope was suitable for examining boron nutrition across horticulture, plant physiology, and fruit-quality research within a single searchable database. Its use provided a consistent basis for literature identification and screening. However, relevant publications not indexed in Scopus may have been missed, potentially affecting the breadth and balance of the evidence considered. This restriction is acknowledged as a limitation of the review. The search was conducted on 21^st^ of April, 2026 and covered publications from 2015 to 2026. The search terms were combined using Boolean operators as follows: (boron OR “boron nanoparticles” OR nanoboron OR “nano-boron”) AND (“fruit quality” OR “post-harvest quality” OR yield OR “horticultural crops”). After screening, studies unrelated to B nutrition in horticultural crops, studies without a focus on fruit growth, yield, fruit quality, or postharvest performance, and non-english articles were excluded, resulting in 172 publications. Of the 172 publications retained after initial screening, 54 publications selected and examined in detail to assess the physiological, biochemical, and mechanistic roles of boron in flowering, fruit set, yield, fruit development, fruit quality, and postharvest performance. This selection was based on the direct relevance to the review questions, availability of quantitative experimental evidence, coverage of different crops and treatment conditions, and representation of contrasting responses. This selection aimed to limit repetitive descriptions of similar mechanisms and responses and was based on author judgment rather than an exhaustive synthesis of all eligible publications. Further, duplicate records were identified by comparing DOIs and, where DOIs were unavailable, article titles, author names, and publication years. Only one record of each publication were retained. Information was extracted from the selected studies on crop species and cultivar, initial B status, B source and formulation, application method and timing, dose, growing conditions, measured physiological, yield, fruit-quality and post-harvest responses, and proposed mechanisms. Particular attention was given to distinguishing responses to B applied alone from those obtained under combined nutrient treatments and to differentiating conventional B from nano-B formulations. The evidence was critically evaluated by comparing the consistency and direction of responses across crops, cultivars, B sources, doses, application methods, and environmental conditions. Established physiological functions were distinguished from mechanisms proposed on the basis of observed changes in growth, yield, biochemical attributes, or postharvest traits. Where findings were inconsistent, crop-specific responses, dose dependence, experimental conditions, and limitations of the available evidence were considered. This approach was used to identify areas of consistent evidence, conflicting findings, and knowledge gaps in B nutrition and fruit quality. The 2015–2026 publication window applied to the Scopus search. Additional publications published before 2015 were identified through backward citation tracking and included for their relevance to boron-mediated reproductive development, yield, and fruit quality. These earlier publications contributed both physiological context and experimental evidence to the narrative synthesis.

## 3 Effect of boron on flowering and fruit set

The reproductive response to boron is closely associated with its availability to actively developing floral tissues and its involvement in processes that support pollen performance and fertilization. Because boron is relatively immobile in many plant species, its supply and timing during the pre-flowering and flowering stages can be particularly important for maintaining adequate B status in floral buds and reproductive tissues. The following evidence highlights the roles of B in pollen germination and tube growth, carbohydrate delivery to reproductive tissues, and subsequent fruit set and retention, while also demonstrating substantial variation among crops and application regimes.

Boron mobility within plants varies among species. Although B is transported mainly through the xylem and has limited phloem mobility in many horticultural crops, species capable of forming B–polyol complexes can redistribute B more effectively to reproductive tissues. Such differences may influence B supply to floral buds, flowers, and developing fruits and partly explain variation in reproductive responses among crops. Therefore, the effectiveness of foliar B application depends on crop-specific B mobility, as well as application dose and timing; limited redistribution may require application during critical reproductive stages (Alvarez-Herrera et al., 2025).

At the cellular level, boron is essential for maintaining cell wall integrity and membrane stability. It forms borate-diol ester linkages with rhamnogalacturonan II (RG-II), contributing to pectin network formation and cell wall stability. This function is particularly important in pollen tubes, where rapid elongation requires continuous cell wall synthesis. During pollen germination, adequate B supply supports vesicle trafficking, cell wall formation, and sustained pollen tube growth, thereby contributing to successful fertilization (Aasim et al., 2022). B also contributes to carbohydrate transport and assimilate partitioning, although the mechanisms and extent of B mobility and redistribution vary among plant species.

Experimental evidence generally supports a positive relationship between B supply and pollen performance, although responses differ according to crop and treatment conditions. In *Pyrus pyrifolia*, foliar application of boric acid at 300 mg L^–1^ significantly enhanced pollen germination, pollen tube growth, anther weight, pollen mass, and B concentration in flower buds (Lee et al., 2009). Similarly, combined soil and foliar application of B increased pollen viability to 71.8% in strawberry (Saridas et al., 2021). These studies provide direct evidence that adequate B availability can improve reproductive traits; however, they represent specific cultivars and application regimes, and therefore their findings cannot necessarily be generalized to all horticultural crops.

A positive association between B concentration in reproductive tissues and fruit set, fruit retention, and yield has been observed in avocado, pomegranate, and macadamia (Boldingh et al., 2016; Davarpanah et al., 2016; De Silva et al., 2022). These observations indicate a relationship between tissue B status and reproductive performance but do not by themselves establish a causal effect of B. In contrast, experimental B application studies provide stronger evidence of a direct response. In macadamia, pre-flowering B application improved fruit set within three weeks of anthesis, while higher B doses increased yield. Similarly, application of B at 0.6 kg ha^–1^ in gulupa (*Passiflora edulis* f. *edulis*) significantly increased flower-bud formation, fruit number, and fruit-set percentage (Quiroga-Ramos et al., 2018). Together, these findings suggest that maintaining adequate B availability before and during flowering can support reproductive success. However, differences in B source, dose, timing, and application method limit direct quantitative comparisons among studies.

Additional evidence from sweet cherry, mango, date palm, papaya, and pomegranate generally supports a beneficial role of B in reproductive development, although responses vary with crop, application timing, dose, and treatment combination. In sweet cherry, pre-bloom B application increased endogenous B concentration and fruit set, while B application in mango and papaya was associated with improved pollen viability, fruit set, pollination, fertilization, and carbohydrate metabolism (Michailidis et al., 2023; Naser et al., 2021; Modi et al., 2012; Muengkaew et al., 2018). In date palm, boric acid applied with pollen grain increased fruit set, fruit weight, and yield, although the specific contribution of B cannot be separated from the combined treatment (Shareef, 2016). Similarly, foliar application of Zn + B in pomegranate increased yield from 13.8 to 18.5 kg tree^–1^ and fruit number from 50.6 to 65.9 fruits tree^–1^ compared with the control (Davarpanah et al., 2016). Overall, these findings suggest that adequate B supply during reproductive stages supports pollen function and fruit set; however, B-only studies provide stronger evidence for B-specific effects, whereas combined treatments mainly demonstrate potential agronomic benefits or nutrient interactions.

The reproductive response to nano-B appears promising but remains insufficiently established. In pomegranate, combined application of nano-Ca (2%) and nano-B (20 ppm) increased yield by 72.9% and 75.41% over two successive seasons; however, because nano-Ca and nano-B were applied together, the specific contribution of nano-B cannot be separated (Abdelwahed et al., 2023). Similarly, foliar application of calcium and boron has been evaluated in pomegranate, with reported effects on fruit quality, yield, and leaf mineral nutrition (Korkmaz and Askın, 2015). In date palm, application of nano-B (0.05%) in combination with wheat seed sprout extract increased bunch weight and yield (Refaai, 2014). Evidence from peach has also indicated beneficial effects of nano-B application on reproductive performance (Al-Dulaimi and Abed, 2025).

Both inadequate and excessive B supply can impair reproductive development, highlighting the narrow range between B deficiency and toxicity. While deficiency and excess may both adversely affect pollen germination and tube growth, the magnitude and threshold of these responses vary among crops and cultivars. In apple (*Malus domestica*), elevated B concentrations shortened pollen tubes and caused tip swelling, whereas very high concentrations markedly reduced pollen germination, demonstrating the dose-dependent nature of B effects (Fang et al., 2016; Hapuarachchi et al., 2022). These responses emphasize the importance of maintaining crop-specific optimum B levels rather than assuming that increased B supply is universally beneficial (**Figure 1**). Moreover, although nano-B has shown potential to improve reproductive and yield-related traits in some crops, evidence remains limited and insufficient to establish its superiority over conventional B sources.

**Figure 1:**
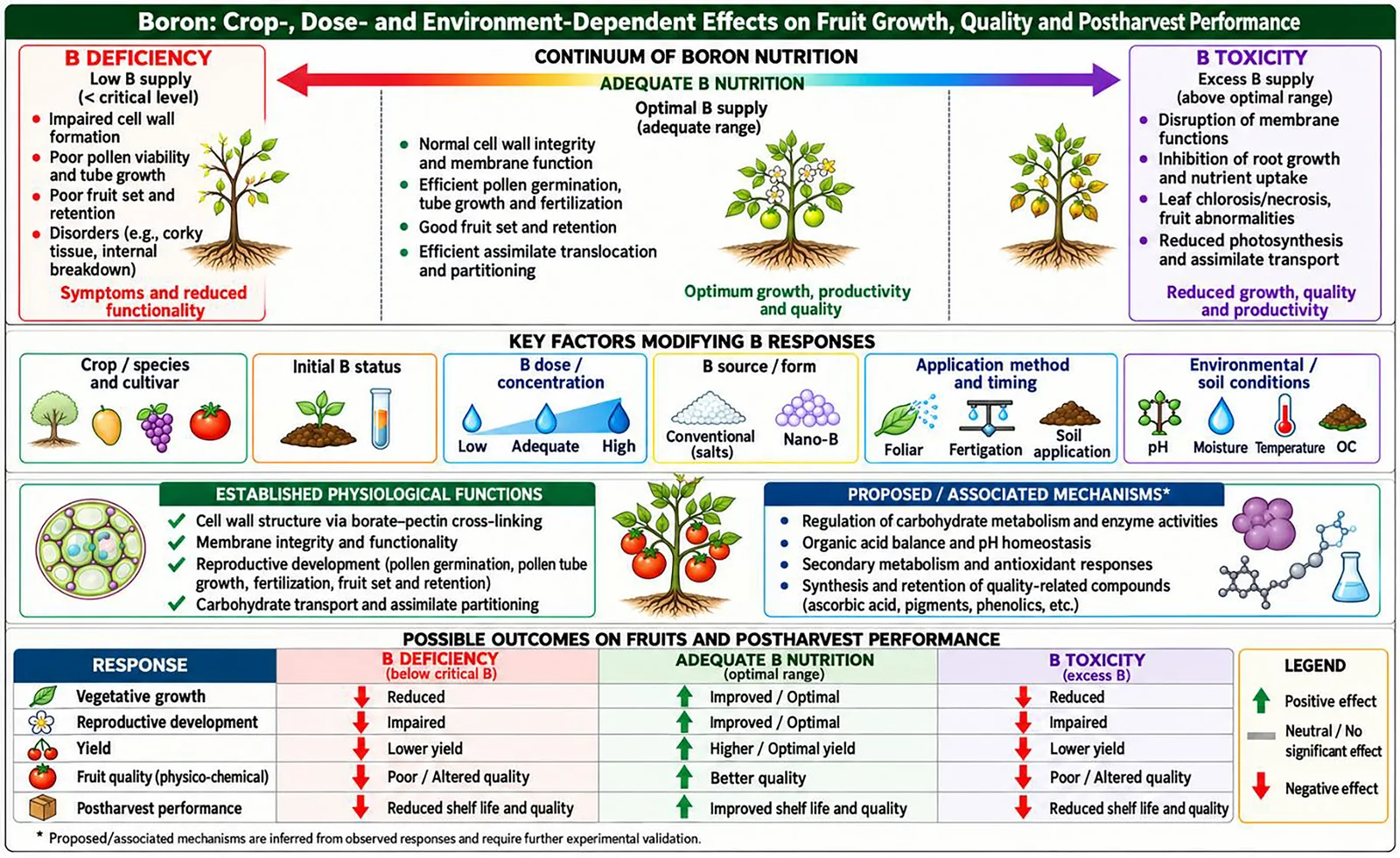
Conceptual framework illustrating the crop, dose, and environment-dependent effects of boron nutrition on fruit growth, yield, quality, and postharvest performance.

However, responses vary with crop species, cultivar, B status, source, dose, application timing, and environmental conditions, while the superiority of nano-B over conventional B remains insufficiently established. Further multi-cultivar and multi-season field studies using standardized B doses and direct comparisons among B sources are required to define optimum B ranges and clarify crop-specific responses.

## 4 Effect of boron on fruit development and post-harvest physical quality

Fruit quality is a key determinant of consumer preference and marketability and includes attributes such as physiological loss in weight (PLW), firmness, color, appearance, size, weight, and volume. Boron (B) contributes to fruit development and quality through its roles in cell-wall stability, membrane integrity, and assimilate transport (Shireen et al., 2018; Alvarez-Herrera et al., 2025). However, responses vary with crop, cultivar, B status, dose, source, application method, timing, and interactions with other nutrients. Therefore, direct B effects should be distinguished from responses obtained with combined nutrient treatments, while proposed mechanisms should be interpreted cautiously.

### 4.1 Physiological loss in weight

Physiological loss in weight (PLW) generally increases during storage, primarily due to moisture loss through transpiration and respiration. Boron application has been reported to reduce PLW in some horticultural crops; however, the magnitude and consistency of the response depend on crop, cultivar, B source, dose, and application conditions. Therefore, the observed reduction in PLW should be interpreted as an improvement in post-harvest water retention rather than attributed to a specific membrane-level mechanism unless supported by direct physiological evidence.

Direct experimental evidence indicates that B application can reduce post-harvest weight loss in some fruits. Immersion of peach fruits in 6 μg mL^–1^ B reduced mass loss from 18% in untreated fruits to 6% (Thomidis and Exadaktylou, 2010), while pre-storage treatment of pear fruits with 3% boric acid reduced weight loss from 5.4% to 3.2% (Kaur et al., 2019). These results provide direct evidence that B treatment can improve weight retention under the tested conditions.

Other studies involved combined nutrient treatments and therefore cannot be interpreted as B-specific effects. In mango, foliar application of Ca + B at 1 mL L^–1^ reduced post-harvest mass loss, while combined application of 0.1% boric acid + 0.2% calcium reduced mass loss to 10.2% (Muengkaew et al., 2018). Although these responses may indicate complementary effects of B and Ca on tissue integrity and water retention, the independent contribution of B cannot be determined from these treatments. Thus, evidence for reduced PLW is stronger for B applied alone under specific experimental conditions, whereas combined B + Ca treatments should be interpreted as overall treatment responses rather than B-specific effects.

### 4.2 Fruit firmness

B can influence fruit firmness through its established role in cell-wall structure, particularly through borate cross-linking of rhamnogalacturonan-II (RG-II). However, the magnitude and direction of the response depend on crop, B status, dose, and treatment conditions. B has also been associated with changes in cell-wall-degrading enzymes during ripening, but such responses should be interpreted as crop- and treatment-specific rather than as universal mechanisms of B-mediated firmness (Montecchiarini et al., 2021; Zhang et al., 2024).

Experimental studies provide evidence of improved firmness following B application in some fruits. In strawberry, B treatment reduced polygalacturonase and pectate lyase activities by 20.2% and 38.1%, respectively, while increasing pectin methyl esterase activity by 18.9%, changes associated with delayed softening (Zhang et al., 2024; Kobra et al., 2017; Mohebbi et al., 2020; Gholamnejad et al., 2023). Similar improvements in firmness were reported in peach and blueberry (Thomidis and Exadaktylou, 2010; Merino-Gergichevich et al., 2016). In mango, 250 mg L^–1^ B increased firmness from 29.0 to 36.7 kg cm^–2^, whereas combined application of 250 mg L^–1^ B + 50% nano-Ca produced 39.4 kg cm^–2^ (El-Motaium et al., 2025). Because the latter treatment combined B with nano-Ca, its higher firmness cannot be attributed specifically to B and may reflect nutrient interaction or treatment complementarity. Similarly, the improved firmness observed following combined B + Ca application in cherry tomato represents an overall treatment response rather than a B-specific effect (Islam et al., 2016).

Nano-B has also shown potential to improve firmness. In peach cv. Florida Prince, foliar application of B nanoparticles at 500 and 1000 ppm increased fruit firmness, possibly through improved B–pectin interactions and cell-wall stability (Al-Dulaimi and Abed, 2025). However, responses were not uniformly positive, as B concentrations above the optimum level were associated with reduced firmness in blueberry (Merino-Gergichevich et al., 2016). Thus, the available evidence indicates that B effects on firmness are dose-dependent and crop-specific, with benefits occurring within an appropriate nutritional range rather than increasing continuously with B supply.

### 4.3 Fruit color and appearance

B may influence fruit color and appearance through effects on cell-wall integrity, phenolic metabolism, and pigment accumulation. Proposed mechanisms include effects on phenylalanine ammonia-lyase, anthocyanin synthesis, membrane stability, and oxidative processes; however, the direct contribution of these pathways to color development is not always demonstrated experimentally (Vera-Maldonado et al., 2024; Dong et al., 2022; Parthiban et al., 2021).

Experimental studies have reported changes in color-related traits following B application, although responses differ among crops. In apple, B application increased fruit color intensity, while in pear it promoted greener fruit color (Wojcik et al., 2008; Kaur et al., 2019). In sweet cherry and pomegranate, B fertilization increased anthocyanin accumulation, rind color, and visual quality (Naser et al., 2021; Michailidis et al., 2023). In contrast, improved color attributes in strawberry following combined B + Ca application represent a treatment response that cannot be attributed specifically to B (Singh et al., 2007). Although changes in photosynthesis, assimilate availability, and phenolic metabolism have been proposed as possible explanations for these responses, the available studies do not consistently establish these pathways as causal mechanisms. Overall, B appears capable of modifying fruit color in some crops, but the direction and magnitude of the response depend on crop, cultivar, maturity stage, B dose, application timing, and associated nutrients.

### 4.4 Fruit size, weight and volume

B availability can influence fruit size, weight, and volume through its established roles in cell-wall formation, cell expansion, reproductive development, and assimilate partitioning (Shireen et al., 2018). However, the magnitude of these responses depends on B status, dose, crop, and application conditions, and greater responses from combined nutrient treatments should not automatically be interpreted as evidence of synergism.

Comparative evidence suggests that nano-B does not consistently outperform conventional B. In olive, nano-chelated B and boric acid applied at equivalent elemental B doses produced trait-specific responses, with nano-B improving fruit and oil yield efficiency, while boric acid resulted in higher oil concentration and altered fatty-acid composition (Vishekaii et al., 2019). In mango, nano-B increased fruit number, fruit weight, and yield under the tested conditions (Abdelaziz et al., 2019); however, these findings represent crop-specific evidence and do not establish general superiority of nano-B.

In mango cv. Zebda, 250 mg L^–1^ B increased fruit volume by 130% compared with the control, indicating a positive response to B applied alone (El-Motaium et al., 2025). Conversely, B deficiency can markedly restrict fruit development. In cape gooseberry, deficiency reduced fruit diameter and weight by 56% and 86%, respectively, and was accompanied by stem-end rot and calyx abnormalities (Martinez et al., 2008; Seo et al., 2022). These findings indicate that adequate B availability is important for normal fruit development, but do not imply that increasing B supply continuously increases fruit size or weight.

Several studies have evaluated B together with other nutrients and therefore provide evidence of treatment-level responses rather than independent B effects. In strawberry cv. Fortuna, combined Ca + B application increased fruit length, diameter, and weight by 21.0%, 35.2%, and 57.1%, respectively, while nano-B + nano-Cu increased fruit weight and total yield by 30.85% and 58.65%, respectively, in strawberry cv. Camarosa (Homed et al., 2026). In mango cv. Zebda, combined application of 250 mg L^–1^ B + 250 mg L^–1^ nano-Ca produced the maximum fruit volume of 698 cm³ fruit^–1^, representing a 178% increase over the control, compared with a 130% increase with 250 mg L^–1^ B alone (El-Motaium et al., 2025). Similarly, combined Ca + B application increased fruit volume in mango (Muengkaew et al., 2018). These results demonstrate the potential value of combined nutrient management, but the additional response cannot be attributed specifically to B unless the individual and interactive effects are experimentally separated.

Overall, the evidence indicates that B availability is important for normal fruit development, but responses are crop- and dose-dependent. B-only studies provide stronger evidence for an independent B effect, whereas combined nutrient treatments primarily demonstrate the potential of integrated nutrient management or nutrient interactions. The available evidence therefore does not support a uniform or continuously increasing effect of B on fruit size, weight, or volume.

## 5 Chemical properties of fruit

Chemical properties such as total soluble solids (TSS), titratable acidity (TA), pH, sugars, organic acids, phenolic compounds, and antioxidant-related enzymes are important determinants of fruit flavor, nutritional value, maturity, and postharvest quality (**Table 1**). Studies have reported changes in these attributes following B application, but the magnitude and direction of the responses vary with crop, cultivar, B status, dose, source, and application conditions. Changes in TSS and acidity may reflect alterations in carbohydrate and organic-acid metabolism, while changes in enzymes such as polyphenol oxidase (PPO) and peroxidase (POD) may be associated with ripening and oxidative processes. However, these mechanisms are not necessarily demonstrated directly in all studies and should therefore be considered proposed explanations rather than universal effects of B. Accordingly, changes in individual chemical attributes should not automatically be interpreted as improvements in fruit quality, as their effects on flavor, nutritional value, and marketability depend on crop-specific quality requirements.

**Table 1:** Representative crop- and dose-dependent responses to boron application in horticultural crops, including positive, neutral, contrasting and adverse responses.

Crop/cultivar	B source and dose	Treatment type	Trait evaluated	Response/observation	Reference
Apple cv. Jonagold	Soil: 2 g B tree^–1^; foliar: 0.03% B	B-only	Fruit set, yield, color	Yield increased by 38% with soil B and 119% with foliar B; fruit color intensity increased	Wojcik et al., 2008
Pear cv. Patharnakh	3% boric acid, pre-storage	B-only	PLW, color retention	Mass loss decreased from 5.4% to 3.2%; greener color retained during storage	Kaur et al., 2019
Peach cv. Andross	B dip, 6 μg mL^–1^	B-only	Firmness, PLW	Firmness increased by 36.9%; mass loss decreased from 18% to 6%	Thomidis and Exadaktylou, 2010
Sweet cherry cv. Skeena	Boric acid, 0.2%	B-only	Fruit set, color	Fruit set and anthocyanin content increased	Michailidis et al., 2023
Avocado cv. Hass	Soil B, 15 or 30 g tree^–1^	B-only	Fruit size, postharvest quality	Fruit diameter increased by about 2%; postharvest quality improved	Hapuarachchi et al., 2022
Mango cv. Zebda	B, 250 mg L^–1^	B-only	Fruit weight, firmness	Fruit weight increased by 128%; firmness increased significantly	El-Motaium et al., 2025
Mango cv. Zebda	B, 250 mg L^–1^	B-only	Fruit volume	Fruit volume increased by 130% over control	El-Motaium et al., 2025
Mango cv. Zebda	B 250 mg L^–1^ + nano-Ca 250 mg L^–1^	Combined B + nano-Ca	Fruit weight, firmness, volume	Combined treatment produced greater responses than B alone for some traits; individual contribution of B should be interpreted cautiously	El-Motaium et al., 2025
Pomegranate cv. Wonderful	Nano-Ca 2% + nano-B 20 ppm	Combined nano-B + nano-Ca	Fruit weight, yield	Fruit weight reached 350–355 g; yield increased by 72.9% and 75.41% in two seasons	Abdelwahed et al., 2023
Date palm cv. Zaghloul	Nano-B 0.05% + wheat sprout extract 1%	Combined nano-B + extract	Fruit weight, yield	Fruit weight increased to 33.5 g compared with 27.0 g in control	Refaai, 2014
Strawberry cv. Chandler	CaCl_2_ + boric acid	Combined B + Ca	Yield, firmness, color	Yield increased by 20%; firmness and color parameters improved	Singh et al., 2007
Cherry tomato cv. Unicorn	B 4.85 mM + CaCl_2–_1 M	Combined B + Ca	Firmness, weight loss	Lower weight loss and greater firmness during storage	Islam et al., 2016
Pecan cv. Wichita	Foliar B: 0, 0.5, 1.0, 1.5 kg B ha^–1^	B-only; contrasting doses	Nut number, yield, kernel weight, nut quality	No significant effect on nut number, yield or nut quality; 1.5 kg B ha^–1^ increased leaf necrosis, indicating possible toxicity	Khalil et al., 2011
Pineapple cv. Comte de Paris	Increasing B doses	B-only; contrasting doses	TSS, fruit quality	Lower doses improved/maintained quality, whereas 4 mg kg^–1^ reduced TSS and negatively affected quality	Wei et al., 2018
Blueberry cvs. Brigitta and Legacy	Increasing B doses	B-only; contrasting doses	TSS, firmness	Moderate B improved TSS/firmness, whereas higher doses reduced these responses	Merino-Gergichevich et al., 2016
Strawberry cv. Chandler	Ca + B	Combined B + Ca	TSS, firmness	TSS was slightly lower than control (7.2 vs. 7.8%), although treated fruits remained firmer during storage	Singh et al., 2007
Apple – cultivar not reported	Elevated B concentrations	B-only; excessive B	Pollen germination/tube growth	Higher B concentrations shortened pollen tubes and caused tip swelling; very high concentrations markedly reduced pollen germination	Fang et al., 2016

### 5.1 Total soluble solids

Total soluble solids (TSS) are an important indicator of fruit sweetness, maturity, flavor, and consumer acceptability. Adequate B availability can influence TSS through its role in carbohydrate metabolism and the translocation of photoassimilates from source leaves to developing fruits. Boron is also associated with the activity of enzymes involved in sugar metabolism, including invertases and sucrose synthases, which may influence sugar accumulation within fruit tissues (Moradinezhad and Ranjbar, 2024). Its contribution to pectin synthesis, cell-wall development, and membrane integrity may additionally affect fruit water balance and maturity. These mechanisms provide a physiological basis for the association between B nutrition and TSS; however, the relative contribution of each process under different crop conditions remains difficult to distinguish.

Several studies have reported increased TSS following B application. In pomegranate, foliar B application increased chlorophyll content, soluble carbohydrates, relative water content, and proline accumulation, contributing to improved fruit quality (Abdelwahed et al., 2023). In cape gooseberry, omission of B reduced °Brix by nearly 40% compared with B-supplied plants, demonstrating the importance of adequate B nutrition for TSS and fruit quality (Martinez et al., 2008). In tomato, boric acid at 5.7 mg L^–1^ increased TSS to 5.13°Brix compared with 4.63°Brix in untreated fruits (Xu et al., 2021). Similarly, B application increased TSS in mango, apple, pineapple, and blueberry (Muengkaew et al., 2018; Wojcik et al., 2008; Wei et al., 2018; Merino-Gergichevich et al., 2016). In pear, postharvest dipping with 3% boric acid delayed the initial increase in TSS during storage but resulted in higher final TSS and better eating quality after storage (Kaur et al., 2019).

The response, however, is not consistently positive and appears to depend on B concentration and treatment conditions. Higher B concentrations negatively affected fruit quality in pineapple and blueberry (Wei et al., 2018; Merino-Gergichevich et al., 2016). In strawberry, combined calcium and B treatment produced slightly lower TSS (7.2%) than the control (7.8%), although treated fruits remained firmer during storage; the lower TSS was associated with delayed ripening and senescence (Singh et al., 2007). In tomato, foliar application of nano-B also produced a concentration-dependent response in fruit TSS, with values varying across nano-B concentrations (Rajani et al., 2022). These findings indicate that increased TSS should not be considered an inevitable response to B application; rather, the direction and magnitude of the response may depend on crop, genotype, B source, dose, formulation, and application conditions. Nano-B has also been investigated for its effects on TSS, with responses varying among crops and treatment combinations. In pomegranate, nano-B at 20 mg L^–1^ increased TSS from 13.50 to 15.00% in the first season and from 13.80 to 15.50% in the second season compared with the control, indicating a positive response to nano-B applied alone (Abdelwahed et al., 2023). In mango cv. Keitte, nano-B increased TSS compared with conventional B treatments under the tested conditions (Abdelaziz et al., 2019). However, comparative evidence from olive using equivalent elemental-B doses showed trait-specific responses to nano-B and conventional B, indicating that the higher TSS observed with nano-B in mango should be interpreted as a crop- and formulation-specific response rather than evidence of consistent superiority (Vishekaii et al., 2019).

In contrast, the highest TSS in pomegranate (17.20 and 17.80%) was obtained with combined nano-Ca + nano-B, which cannot be attributed specifically to nano-B because the treatment contained both nutrients (Abdelwahed et al., 2023). Similarly, TSS responses reported for some strawberry and other crops involved nano-B in combination with additional nutrients and should therefore be interpreted as overall treatment responses rather than independent nano-B effects. Overall, the available evidence indicates that nano-B can influence TSS under some conditions, but responses are crop-, dose-, and treatment-dependent, and higher responses from combined nano-nutrient treatments should not be interpreted as evidence of a specific nano-B effect.

### 5.2 Titratable acidity and fruit pH

Titratable acidity (TA) and fruit pH are important indicators of organic acid status and contribute to fruit flavor, maturity assessment, consumer acceptance, and postharvest behavior. Studies have reported changes in TA and pH following B application, but the direction and magnitude of these responses vary with crop, cultivar, B status, dose, maturity stage, and treatment conditions. B has established roles in cell-wall and membrane integrity; however, direct evidence that B universally regulates organic-acid synthesis, compartmentalization, retention, or degradation is limited. Changes in TA or pH following B application may therefore reflect alterations in organic-acid metabolism or tissue development, but these mechanisms should be considered proposed explanations unless directly demonstrated experimentally. In addition, higher or lower acidity cannot be universally regarded as an improvement in fruit quality because the desirable acidity depends on crop-specific maturity, sugar–acid balance, and sensory characteristics.

Several studies have reported that B application can maintain or increase fruit acidity. In pear, B treatment enhanced TA retention by 22.8–40.0% compared with untreated fruits during storage (Kaur et al., 2019). In apple, foliar and soil application of B increased TA by 14.2% and 3.1%, respectively, compared with the control (Wojcik et al., 2008). In tomato, foliar and soil application of boric acid at 1.90–5.70 mg L^–1^ increased TA by up to 8.90% (Xu et al., 2021). Similarly, B application at 2 g L^–1^ increased acidity in bell pepper, while foliar B increased TA by 3% in strawberry (Pagard et al., 2024; Singh et al., 2007). In strawberry cv. Fortuna, integrated nutrient management combined with foliar calcium and B at 4 mL L^–1^ resulted in the highest TA of 0.82%, compared with 0.61% under the lowest treatment (Mohamed et al., 2021). These findings generally support a positive role of adequate B nutrition in maintaining fruit acidity, although the magnitude of the response differs among crops and treatment conditions.

In contrast, B application does not invariably increase acidity. In mango cv. Zebda, foliar application of 250 mg L^–1^ boron + 250 mg L^–1^ nano-calcium reduced total acidity by 72% compared with the control (El-Motaium et al., 2025). This reduction may be associated with differences in fruit maturity or sugar–acid balance; however, the treatment response cannot by itself be interpreted as improved palatability in the absence of sensory evaluation.

However, crop-specific differences were evident, as boron application at 0.5 to 4.0 mg kg^–1^ had no significant effect on TA in pineapple (Wei et al., 2018). This contrasting response indicates that B may either promote the retention of organic acids or be associated with reduced acidity depending on crop, maturity stage, dose, and interactions with other nutrients. Thus, increased TA should not be considered a universal indicator of a beneficial B response.

Fruit pH also exhibits crop-specific responses. In cape gooseberry, B application did not significantly affect fruit pH, indicating limited responsiveness under the conditions studied (Martinez et al., 2008). In contrast, application of borax at 100 g plant^–1^ significantly reduced pH in *Citrus grandis*, indicating increased acidity (Luo et al., 2024). Conversely, combined application of 120 mg L^–1^ nano-zinc and 3.25 mg L^–1^ nano-B increased pomegranate juice pH by 17.5%, from 3.42 to 4.02 (Davarpanah et al., 2016). These contrasting responses demonstrate that B does not produce a consistent directional change in fruit pH across crops.

Nano-B has also been investigated for its effects on TSS, although comparative evidence remains limited and crop-dependent. In mango cv. Keitte, nano-B increased TSS compared with conventional B treatments under the tested conditions (Abdelaziz et al., 2019). However, comparative evidence from olive using equivalent elemental-B doses showed that nano-B and conventional B produced trait-specific responses, indicating that higher TSS with nano-B should be interpreted as a crop- and formulation-specific response rather than evidence of consistent superiority (Vishekaii et al., 2019).

Recent studies have also examined nano-boron as a potential source for modifying fruit acidity. In mango cv. Keitte, foliar application of nano-boron at 20 mg L^–1^ resulted in acidity values of 0.83% and 0.82% during the first and second seasons, respectively, compared with conventional boron treatments (Abdelaziz et al., 2019). Similar reductions in fruit acidity following nano-boron application have been reported in pomegranate and strawberry (Davarpanah et al., 2016; Homed et al., 2026). However, comparative evidence from olive using equivalent elemental-B doses showed that nano-B and conventional B produced trait-specific responses (Vishekaii et al., 2019). Therefore, reductions in acidity observed with nano-B should be interpreted as crop- and treatment-specific responses rather than evidence of consistent superiority over conventional B. These findings indicate that nano-B can modify fruit acidity under some experimental conditions; however, whether a reduction in acidity represents improved maturity, flavor, or eating quality depends on the crop, maturity stage, and overall sugar–acid balance. In the absence of sensory measurements, changes in TA should therefore be interpreted as physicochemical responses rather than direct evidence of improved eating quality.

However, the evidence should be interpreted cautiously because the reported responses vary among crops and experimental conditions, and some studies involve nano-boron in combination with other nutrients. Therefore, the available evidence is not yet sufficient to establish that nano-boron consistently reduces fruit acidity or is superior to conventional boron.

### 5.3 Ascorbic acid

Ascorbic acid (AsA) or vitamin C, is important nutritional and antioxidant component of fruits Although boron is not directly involved in the biosynthesis of ascorbic acid but adequate supply of boron is essential for minimizing its degradation and maintaining cellular redox balance (Li et al., 2023). B nutrition may influence ascorbic acid accumulation through effects on plant metabolism and antioxidant responses; however, the specific mechanisms underlying this response remain insufficiently understood (Gad and Sirko, 2024).

Several studies have demonstrated the beneficial effects of boron application on preserving or enhancing ascorbic acid content in fruits. In ‘Patharnakh’ pear, pre-storage treatment with 3% boric acid maintained 48% higher ascorbic acid levels than the untreated control during storage, which was attributed to suppression of polyphenol oxidase (PPO) activity, thereby delaying oxidative deterioration (Kaur et al., 2019). B fertilization also positively influenced vitamin C concentration in pineapple (Wei et al., 2018). In tomato, soil application of B increased AsA by 185% over the untreated control (Xu et al., 2021). Similarly, B supplementation increased vitamin C and anthocyanin contents in pomegranate, while foliar borax at 0.9% increased AsA in ber (Abdelwahed et al., 2023; Meena et al., 2008).

B deficiency can have the opposite effect by increasing oxidative stress and membrane damage. Excess reactive oxygen species may promote lipid peroxidation and accelerate the oxidation of AsA to dehydroascorbic acid, thereby reducing vitamin C concentration and antioxidant capacity (Shireen et al., 2018; Li et al., 2023). This supports the importance of adequate B supply for maintaining AsA during fruit development and storage. Recent studies have also investigated nano-boron as a potential source for modifying ascorbic acid accumulation. Recent studies have also investigated nano-boron as a potential source for modifying ascorbic acid accumulation. In mango cv. Keitte, foliar application of nano-B at 20 ppm resulted in higher ascorbic acid content, 48.3 and 48.0 mg 100 mL^–1^ juice during the first and second seasons, respectively, than the conventional B treatments (Abdelaziz et al., 2019). Similarly, nano-B application increased vitamin C content in broccoli heads compared with conventional boric acid fertilizer (Shams and Abbas, 2019). These findings indicate that nano-B may influence AsA accumulation under specific crop and treatment conditions. However, the available evidence is limited to a small number of crops and formulations, and comparative studies directly evaluating nano-B against conventional B remain scarce. Therefore, the potential advantage of nano-B for AsA accumulation should be considered promising but not yet conclusive.

### 5.4 Total sugars

Total sugars contribute to fruit sweetness, flavor, maturity, and consumer acceptance. Boron is associated with carbohydrate transport and accumulation, while B deficiency may restrict carbohydrate translocation to developing fruits (Thakur et al., 2023; Ewais et al., 2020). Studies in ber have reported increased total, reducing, and non-reducing sugars following B application (Meena et al., 2008). However, responses from combined treatments, such as Ca + B in strawberry, nano-Zn + nano-B in pomegranate, and B + nano-Ca in mango, represent overall treatment effects and cannot be attributed specifically to B (Mohamed et al., 2021; Davarpanah et al., 2016; El-Motaium et al., 2025).

Importantly, nano-B responses may be non-linear and dose-dependent. In strawberry cv. Camarosa, 10 mg L^–1^ nano-B increased total and reducing sugars by 46.9% and 70.1%, respectively, whereas 20 mg L^–1^ produced smaller increases of 22.8% and 37.1% (Homed et al., 2026). Thus, a higher nano-B concentration did not produce a proportionally greater response. The combined nano-B + nano-Cu treatment produced the highest sugar contents, but this should be regarded as a combined-treatment response rather than evidence of synergy unless a significant interaction was experimentally demonstrated. Crop-specific studies have reported increases in total sugars following nano-B application in date palm and mango under particular treatment conditions (Refaai, 2014; Abdelaziz et al., 2019). However, these individual observations should not be interpreted as evidence of a generalized effect of nano-B on sugar accumulation.

### 5.5 Total phenolic compounds

Phenolic compounds are important secondary metabolites contributing to the antioxidant capacity, nutritional quality and color development of fruits. Boron may influence phenolic metabolism through effects on carbon partitioning and related metabolic pathways. However, the magnitude and direction of the response vary among crops and treatment conditions. In pomegranate cv. Ardestani, combined application of 120 mg L^–1^ nano-Zn and 6.50 mg L^–1^ nano-B resulted in a small increase in total phenolic content (409 vs. 406.64 mg 100 g^–1^ fresh weight in the control), although the magnitude of this difference was limited (Davarpanah et al., 2016). In contrast, changes in phenolic compounds following B + Zn application have been reported in olive and pungent pepper (Saadati et al., 2016; Manas et al., 2014). Thus, statistically detectable changes in phenolic concentration should be interpreted in relation to their magnitude and biological significance rather than assumed to represent meaningful improvements in fruit quality.

In contrast, direct application of boron to ‘Merlot’ grape bunches altered the phenolic composition of grape tissues, with increases in several phenolic compounds, particularly anthocyanins, phenolic acids, and flavanols, although the magnitude of the response varied with boron concentration and vintage (Bredun et al., 2023). The study also showed greater distribution of polyphenols within grape tissues following boron application, illustrating that phenolic responses to B may differ according to crop species, tissue, application method, concentration, and the specific phenolic compounds evaluated. However, the response of phenolic compounds to boron application is not always consistent and may vary depending on crop species, cultivar, tissue, and nutritional status. For example, in mango cv. Zebda, the combined foliar application of 250 mg L^–1^ boron and 250 mg L^–1^ nano-calcium recorded the lowest total phenol content (20.52 mg kg^–1^), representing a 78% reduction compared with the control (El-Motaium et al., 2025). Similar reductions in total phenol concentration following boron application were reported in olive cv. Frantoio leaves and buds (Hegazi et al., 2018). These contrasting responses further support the need to interpret changes in phenolic concentration within the specific nutritional and experimental context rather than assuming that B application invariably increases or decreases total phenolic content.

The relationship between B nutrition and phenolic accumulation may therefore be context-dependent. Under B deficiency, changes in carbohydrate metabolism and the pentose phosphate pathway may favor the production of precursors for phenolic synthesis, resulting in increased phenolic accumulation as part of a stress response (Hegazi et al., 2018). Consequently, higher phenolic concentration should not automatically be interpreted as evidence of improved fruit quality, because it may also indicate greater metabolic stress. Excessive phenolic accumulation may contribute to enzymatic browning and undesirable color changes. Thus, the available evidence suggests that adequate B may help regulate phenolic metabolism rather than simply increasing total phenolic concentration.

### 5.6 Total carotenoids and flavonoids

Carotenoids and flavonoids contribute to fruit color, antioxidant capacity, and nutritional value (**Table 2**). Boron has been associated with changes in these compounds, although the available evidence is limited and often involves combined nutrient treatments. In mango cv. Zebda, combined foliar application of 250 mg L^–1^ B + 250 mg L^–1^ nano-Ca produced the highest carotenoid (7.32 mg g^–1^) and flavonoid (48.60 mg quercetin equivalent 100 g^–1^ fresh weight) contents, representing increases of 341% and 251%, respectively, over the control (El-Motaium et al., 2025). However, these responses cannot be attributed specifically to B. In broccoli, increasing B application from 0.4 to 1.2 kg fed^–1^ increased carotenoid content from 14.7 to 25.3 mg 100 g^–1^ fresh weight, while B application at 300 mg L^–1^ increased flavonoid accumulation in dragon fruit buds (Metwaly, 2016; Sahu et al., 2022). Overall, the available evidence indicates that B may influence carotenoid and flavonoid accumulation under specific crop, dose, and application conditions, but the limited number of studies and prevalence of combined treatments do not support a generalized B effect.

**Table 2:** Biochemical responses to conventional boron, nano-boron, and combined treatments in horticultural crops, with plant tissue analyzed and proposed interpretations.

Crop/cultivar	Application method	B/nano-B treatment	Treatment type	Plant tissue analyzed	Major biochemical response	Proposed mechanism/interpretation (where applicable)	Reference
Pear cv. Patharnakh	Pre-storage dip	3% boric acid	Conventional B alone	Fruit	Ascorbic acid maintained at a higher level; PPO activity was reduced during storage	May be associated with delayed oxidative deterioration and reduced PPO activity	Kaur et al., 2019
Cape gooseberry (Physalis peruviana)	Foliar application/nutrient treatment	3 mg L^–1^ B	Conventional B alone	Fruit	B supply increased AsA; B omission reduced °Brix by nearly 40%	May indicate a role of adequate B nutrition in carbohydrate and antioxidant metabolism	Martinez et al., 2008
Tomato (*Solanum lycopersicum*)	Foliar spray	Boric acid, 1.90–5.70 mg L^–1^	Conventional B alone	Fruit	TSS increased to 5.13°Brix; TA and AsA also increased with B application	May reflect changes in carbohydrate and organic-acid metabolism under adequate B supply; specific pathways were not directly demonstrated	Xu et al., 2021
Ber cv. Gola	Foliar spray	Borax, 0.9%	Conventional B alone	Fruit	Total, reducing and non-reducing sugars and AsA increased	May be associated with altered carbohydrate accumulation and antioxidant metabolism	Meena et al., 2008
Apple (*Malus domestica*)	Soil and foliar application	2 g B tree^–1^ soil; 0.03% B foliar	Conventional B alone	Fruit	TA and fruit color intensity increased	May be associated with effects of adequate B nutrition on fruit development and organic-acid metabolism	Wojcik et al., 2008
Sweet cherry (*Prunus avium*)	Pre-bloom application	Boric acid, 0.2%	Conventional B alone	Fruit	Anthocyanin content and fruit set increased	May be associated with changes in developmental and pigment-related metabolism; causal pathways were not directly established	Michailidis et al., 2023
Dragon fruit	Foliar application	300 mg L^–1^ B	Conventional B alone	7-day-old flower buds	Flavonoids and other bioactive compounds increased; enzyme activities related to carbohydrate and cell-wall metabolism were also affected	May indicate modification of secondary and carbohydrate metabolism under the tested B supply	Sahu et al., 2022
Pineapple (*Ananas comosus*)	B application	0.5–4.0 mg kg^–1^ B	Conventional B; contrasting dose response	Fruit	Higher B concentrations were associated with reduced TSS/fruit quality	Indicates a crop- and dose-dependent response; excessive B may adversely affect biochemical quality	Wei et al., 2018
Blueberry cvs. Brigitta and Legacy	Foliar spray	Different B concentrations	Conventional B; contrasting doses	Fruit	Moderate B improved some quality traits, whereas higher concentrations reduced responses	Demonstrates dose-dependent biochemical and quality responses to B	Merino-Gergichevich et al., 2016
Pecan cv. Wichita	Foliar spray	Boric acid (H_3_BO_3_), 50 and 100 mg L^–1^	Conventional B alone	Leaflets	B application increased SOD and GPx activity and affected antioxidant capacity, CAT activity, phenols, flavonoids and anthocyanins	Indicates that conventional B can modify oxidative metabolism and bioactive-compound responses in leaves; responses were dose-dependent	Cruz-Alvarez et al., 2024
Broccoli (*Brassica oleracea* var. *italica*)	Foliar application	Nano-B and conventional B	Comparative conventional B vs nano-B	Heads and shoots	Nano-B produced a greater vitamin C response than conventional B under the reported conditions; P and B concentrations also differed between treatments	Indicates a formulation-specific response; does not establish universal superiority of nano-B	Shams and Abbas, 2019
Mango cv. Keitte	Foliar spray	20 mg L^–1^ nano-B	Nano-B alone	Fruit	AsA and total sugars increased	Demonstrates a dose-dependent response; underlying metabolic mechanism remains to be established	Abdelaziz et al., 2019
Strawberry cv. Camarosa	Foliar spray	10 and 20 mg L^–1^ nano-B	Nano-B alone; contrasting doses	Fruit	TSS, sugars and vitamin C increased; lower concentration produced greater sugar responses	Demonstrates a dose-dependent response; underlying metabolic mechanism remains to be established	Homed et al., 2026
Peach cv. Florida Prince	Foliar spray	1000 ppm B_2_O_3_ nanoparticles	Nano-B alone	Fruit	TSS increased	May be associated with altered assimilate partitioning; this mechanism was not directly demonstrated	Al-Dulaimi and Abed, 2025
Date palm cv. Zaghloul	Foliar spray	0.1% nano-B	Nano-B alone	Fruit	TSS and total sugars increased	May be associated with altered carbohydrate accumulation or translocation; specific mechanism was not directly demonstrated	Refaai, 2014
Pomegranate cv. Wonderful	Foliar spray	20 mg L^–1^ nano-B	Nano-B alone	Fruit	TSS increased from 13.50 to 15.00% and from 13.80 to 15.50% in two seasons	May reflect changes in solute accumulation and fruit biochemical development; specific causal pathways were not demonstrated	Abdelwahed et al., 2023
Olive cv. Zard	Foliar application	Nano-chelated B vs. boric acid at comparable B doses	Comparative nano-B vs conventional B	Fruit and oil	Nano-B and boric acid produced different trait-specific responses in yield/oil characteristics; antioxidant activity and total phenols were affected	Indicates that nano-B and conventional B may differ in their effects depending on the trait; does not demonstrate consistent nano-B superiority	Vishekaii et al., 2019
Mango cv. Zebda	Foliar application	250 mg L^–1^ B + 250 mg L^–1^ nano-Ca	Combined B + nano-Ca	Fruit	TSS, sugars, carotenoids and flavonoids increased; acidity decreased	Represents a combined nutrient response; the independent contribution of B cannot be separated	El-Motaium et al., 2025
Pomegranate cv. Ardestani	Foliar spray	6.5 mg L^–1^ nano-B + nano-Zn	Combined nano-B + nano-Zn	Fruit	TSS, sugars and phenolic compounds increased; acidity decreased	Represents a combined nano-nutrient response; B-specific effects cannot be separated	Davarpanah et al., 2016
Date palm cv. Zaghloul	Foliar spray	0.05% nano-B + 1% wheat sprout extract	Combined nano-B + extract	Fruit	Fruit/yield responses were reported	The response cannot be attributed specifically to nano-B because wheat sprout extract was also included	Refaai, 2014

## 6 Limitations and research gaps in nano-boron research

Despite the promising physiological and agronomic benefits reported for nano-boron, the current evidence base in horticultural crops remains limited and heterogeneous. Although nano-boron has been associated with improved nutrient acquisition and utilization under certain experimental conditions, direct evidence demonstrating greater nutrient-use efficiency or reduced nutrient losses compared with conventional boron fertilizers remains limited. These potential benefits should also be considered alongside the potentially higher costs associated with nanoparticle synthesis, purification, characterization, formulation, storage, and quality control. Therefore, the economic advantage of nano-boron over conventional boron fertilizers cannot be assumed solely from improved plant performance and requires validation through field-scale cost–benefit analyses and life-cycle assessments (Channab et al., 2024; Ayenew et al., 2025). A major limitation of the current literature is the scarcity of well-replicated, multi-location, and multi-season field studies. Much of the available evidence has been generated under pot, greenhouse, or small-scale experimental conditions, often using single cultivars and specific environmental conditions. Importantly, nano-B is not a uniform treatment category; differences in particle composition, size distribution, morphology, surface properties, formulation, and stability can substantially influence foliar deposition, uptake, mobility, and biological responses. Consequently, results obtained with different nano-B formulations should not be directly generalized or considered equivalent without adequate physicochemical characterization. In addition, relatively few studies have directly compared nano-B with conventional B fertilizers at equivalent elemental B concentrations under field conditions, making it difficult to determine whether reported responses arise from nanoparticle-specific properties or enhanced B availability.

Nanoparticle stability represents another important practical consideration. Aggregation or changes in particle size and surface properties during preparation, storage, or application may influence suspension stability, foliar deposition, uptake, mobility, and nutrient availability. The requirement for specialized synthesis, purification, characterization, formulation, and quality-control facilities may further increase production costs and complicate large-scale implementation. These factors are particularly important when considering the transition from laboratory-scale formulations to commercially consistent products.

Environmental safety also requires greater attention. Information remains limited regarding the persistence, transformation, mobility, and accumulation of nano-B in soil and edible plant tissues, as well as its potential effects on soil microorganisms and other non-target organisms. Importantly, an increase in total tissue B concentration does not by itself demonstrate uptake or persistence of intact nanoparticles, because total B measurements cannot distinguish ionic or dissolved B from particulate or transformed forms. Therefore, studies investigating nanoparticle fate and food safety should incorporate appropriate analytical approaches capable of distinguishing dissolved/ionic B from particulate forms. Such information is essential for assessing the actual fate of nano-B and its potential accumulation in edible tissues.

Potential phytotoxicity is another important concern because boron has a relatively narrow range between deficiency and toxicity. Enhanced B uptake following nano-B application does not necessarily indicate improved plant performance if tissue B concentrations exceed the crop-specific optimum. Therefore, dose–response studies should report initial crop B status and final tissue B concentrations, as the biological response to a given nano-B dose depends on the plant’s pre-existing B status and resulting tissue B concentration. Excess B may cause leaf injury, growth inhibition, oxidative stress, and other toxicity symptoms, with responses varying according to nanoparticle characteristics, concentration, crop species, cultivar, and environmental conditions. Thus, nano-B should not be considered inherently safer than conventional B simply because it can improve nutrient-use efficiency.

Overall, the current evidence on nano-B in horticultural crops remains limited and heterogeneous, with responses varying according to crop and cultivar, B status, nano-B formulation, dose, application method, and environmental conditions. Although several studies have reported beneficial effects on yield and fruit quality, the available evidence is insufficient to establish consistent superiority of nano-B over conventional B fertilizers. Future research should therefore prioritize well-designed field experiments using standardized nano-B formulations and equivalent elemental-B doses, together with direct comparisons with conventional B sources. Greater attention is also needed to dose optimization, uptake and translocation, long-term agronomic performance, environmental safety, and economic feasibility. Such integrated research will help determine the conditions under which nano-B can provide reproducible and practical benefits in horticultural production.

## 7 Conclusion and future perspectives

Boron (B) is an essential micronutrient involved in reproductive development, fruit growth, yield, and fruit quality in horticultural crops. The reviewed evidence indicates that adequate B supply can improve selected yield and quality attributes, whereas both deficiency and excess can adversely affect plant performance. However, responses are strongly influenced by crop and cultivar, initial B status, dose, application method, developmental stage, and environmental conditions, and may be non-linear. Thus, optimum rather than increased B supply is essential for consistent crop responses.

Nano-boron has potential to improve B delivery and nutrient-use efficiency, but the available evidence remains limited and heterogeneous. Interpretation is complicated by differences among nano-B formulations and the frequent use of nano-B with nano-Ca, nano-Zn, nano-Cu, or other nutrients. Such combined treatments cannot establish the independent effect of nano-B, while direct comparisons with conventional B under equivalent field conditions remain limited. Phytotoxicity, nanoparticle stability, environmental fate, food safety, cost, and scalability also require further evaluation.

Future studies should focus on crop-specific dose–response experiments, multi-location and multi-season field trials, and direct comparisons of nano-B and conventional B at equivalent elemental B concentrations. Studies involving combined nutrients should include appropriate single-factor controls and factorial designs to distinguish independent, additive, and interactive effects. Standardized characterization of nano-B and integrated assessment of agronomic performance, safety, environmental fate, and economic feasibility will be essential to determine its practical value in horticultural production.
